# Exploring Community Leaders’ Understanding and Attitudes Toward Depression Among Older Korean Americans

**DOI:** 10.1093/geroni/igaa057.2172

**Published:** 2020-12-16

**Authors:** Eunhye Kim

**Affiliations:** Augusta University, Augusta, Georgia, United States

## Abstract

This study explores community leaders’ understanding and attitudes toward depression among older Korean Americans. The community leaders who provide services for older adults play a significant role because these leaders typically engage with the target population by providing services. A qualitative method was applied for this study. A total of 12 community leaders were interviewed for 60 minutes using the semi-structured interview guide. The constant comparative approach was employed to analyze data using NVivo software. The findings indicated that the community leaders were aware of the severity of the issues, but lacked knowledge regarding depression and had stereotypes against depression. Those leaders identified negative personality issues of people with depression and desired to avoid discussing depression with older adults. The results suggest that interventions are essential to support community leaders and empower them to take actions to provide services to promote understanding of depression in older adults.

